# Physiological and biochemical responses of sugar beet (*Beta vulgaris* L) to ultraviolet-B radiation

**DOI:** 10.7717/peerj.6790

**Published:** 2019-05-03

**Authors:** Parisa Rahimzadeh Karvansara, Seyed Mehdi Razavi

**Affiliations:** Department of Biology, Faculty of Sciences, University of Mohaghegh Ardabili, Ardabil, Iran

**Keywords:** Ultraviolet-B, *Beta vulgaris*, Antioxidant enzymes, Photosynthesis, Proline, Betalain, Oxidative stress

## Abstract

Ultraviolet radiation can cause many serious problems for all living organisms. With a growing population, the UV sensitivity of crop plants presents a particular problem. To evaluate the suitability of growing in areas under UV irradiance, the influence of different doses of UV-B (3.042, 6.084 and 9.126 kJm^−2^d^−1^) on the sugar beet (*Beta vulgaris L*) plants was studied. UV-B induced a significant decrease in growth displayed as reduced height and fresh and dry weight. This reduction is not dose dependent and was associated with diminishing photosynthetic O_2_ evolution, relative chlorophyll content, photosynthetic pigments and chlorophyll fluorescence. On the other hand, antioxidant enzyme activities, total protein content, compatible solutes, total free amino acids and total betalain content were increased under 9.126 kJm^−2^d^−1^ UV-B treatments, representing mechanisms by which the plants coped with the stress. The oxidative stress upon UV-B treatment was evident by increased malondialdehyde (MDA) content, however, hydrogen peroxide (H_2_O_2_) was not affected in UV-B exposed plants. Thus, the studied sugar beet variety BR1seems to be suitable particularly for areas with high doses of UV-B irradiation.

## Introduction

Ultraviolet radiation (UV) with wavelengths between 10 nm and 400 nm constitutes about 10% of the total light output of the Sun. The high energy radiation is mostly absorbed by the oxygen and ozone in the atmosphere. Therefore, reductions in the ozone layer, such as those observed transiently in the 1990s, are connected with increased levels particularly of UV-B, with consequences for human health (Ramanathan & Feng, 2009; Young, Claveau & Rossi, 2017). Current levels of UV-B range from 2 to12 kJm^−2^ per day at different parts of the Earth (Lidon et al., 2012). UV-B radiation can also cause a number of malfunctions and growth inhibition in plants. Many plant processes such as photosynthesis, biomass allocation, dark respiration, transpiration, and ultimately growth are affected by UV radiation. This is a consequence of macromolecule damage by UV-B causing double helix DNA breaks, lipids peroxidation and protein degradation in exposed plants. However, plants can induce defense reactions to UV radiation and may achieve some degree of tolerance (Rahimzadeh, HosseiniSarghein & Dilmaghani, 2011).

Tolerance to UV is different among various species and even closely related genotypes (Singh et al., 2017). One of the most common responses to UV-B is a leaf area decrease because of reduction in both cell division and cell expansion (Wargent et al., 2009). On the other hand, plants display biochemical and phytochemical adaptations to UV-B radiation by enhancing their primary and secondary metabolites to increase protection from UV radiation. One class of secondary metabolites protecting against UV are the phenolic compounds, such as lignin, a cell wall ingredient of all vascular plants and some algae. Cell wall lignification is induced by UV radiation and it is the first defensive barrier of epidermis against UV-B (Barros et al., 2015). Among other phenylpropanoids efficient in UV protection, anthocyanins are the most widespread (Bandurska, Niedziela & Chadzinikolau, 2013). Interestingly, in species of the order Caryophyllales, most plants contain betalains instead of anthocyanins. Betalains are a class of compounds with a nitrogenous core structure which play antioxidant role in plants and in human diet. These two groups of pigments never occur in the same plants, because anthocyanin producing enzymes are not expressed in betalain containing plants (Khan & Giridhar, 2015). It is also not known whether betalains play a role in UV protection in red beets like the anthocyanins.

One of the betalain producing plants is sugar beet (*Beta vulgaris*) which, because of its ability to accumulate a large quantity of sugar in storage roots, provides about 40% of the world’s sugar production. However, environmental factors can reduce sugar production potential by affecting sugar beet plant productivity (Khan, Kumar & Giridhar, 2016). There have been many investigations on response of sugar beet plants to abiotic stresses including drought, heat, cold and UV radiation. Indeed, reductions in leaf area, fresh and dry weights, chlorophyll and carotenoid contents of sugar beet exposed to UV radiation with different light condition back ground have been reported (Panagopoulos, Bornman & Bjorn, 1990). Furthermore, Clarke et al. (1995) showed that UV-B radiation reduced chlorophyll fluorescence in 22 commercial sugar beet varieties. These results showed that the reduction rate was different in all varieties with about a ten-fold difference in the degree of Fv/*F*_m_, reduction between the most sensitive and most tolerant cultivars. Generally, the previous studies concluded that sugar beet is a stress tolerant crop.

However, given the importance of sugar beet for agriculture in Iran, more data are needed about the tolerance of this crop to UV. Since the previous detailed analysis of UV stress in sugar beet used high doses of UV-B, which led to a 77% reduction in leaf area (Khan, Kumar & Giridhar, 2016), data from treatments corresponding to the more physiological doses of UV-B radiation are needed. In the present work we evaluated a widely grown Iranian multigerm cultivar of sugar beet BR1 for its physiological and biochemical responses to three different UV-B radiations to evaluate its potential as an efficient crop in the areas with different doses of UV-B.

## Materials & Methods

### Growth condition and UV-B treatment

The seeds of BR1 cultivar of sugar beet *(Beta vulgaris L.)* (Obtained from Agriculture Organization of West Azerbaijan Province, Urmia, Iran) were sterilized with 10% sodium hypochlorite for 10 min and then soaked in distilled water. After 4 days germination the seedlings were transferred to pots filled with soil. All plants were grown in a growth chamber (Jeiotech GC-3007 LH, Korea) at 25/20 °C (day/night), with a 16 h light/8 h dark photoperiod for 30 days under light intensity of 500 µmol m^−2^s^−1^in PAR region. Afterwards, the plants were divided into 4 groups including control and three experimental groups exposed to 3.042, 6.084 and 9.126 kJm^−2^d^−1^UV-B radiation for one week. UV-B radiation was provided by TL20W/12 RS SLV/25 lamp (Philips, Germany) positioned 20 cm above the plants. The lamp was wrapped in a 0.13 mm cellulose diacetate film and polyethylene foil to absorb radiation below 290 nm (UV-C) and above 320 nm (UV-A), respectively. Intensity of radiation was measured with an ASD Handheld 2 spectro radiometer.

### Growth and leaf relative water content measurement

Plant height, fresh weight and leaf area were measured immediately after removal from the pots. Dry weight was determined after drying at 75 °C for 48 h. The leaf relative water content was determined in the fully expanded youngest leaf of the main shoot. The fresh weight (FW) of the sample leaves was recorded and the leaves were put in dark room in distilled water for 24 h in order to obtain the turgid weight (TW). Then, the samples were dried in an oven at 75 °C to obtain dry weight (DW). Leaf relative water content was calculated using the following formula (Sade, Galkin & Moshelion, 2015):

\begin{eqnarray*}LRWC(\text{%})=[(FW-DW)/(TW-DW)]\times 100. \end{eqnarray*}

### Photosynthetic pigments measurement

In order to measure chlorophyll and carotenoid content, 0.1 g of complete leaves were ground in 1 ml of 80% acetone and centrifuged at 2,700 rpm for 10 min. The supernatant was directly used for spectrophotometric measurements of absorbance at 647, 663 and 470 nm. To estimate chlorophyll a, chlorophyll b, total chlorophyll and carotenoids contents by spectrophotometer, following equations were used (Gross, 1991):

\begin{eqnarray*}\mathrm{Total}~\mathrm{chlorophyll}~(\mathrm{\mu }\mathrm{g/ ml})=(20.21\times {A}_{645})+(8.02\times {A}_{663}) \end{eqnarray*}

\begin{eqnarray*}\mathrm{Chlorophyll}~\mathrm{a}~(\mathrm{\mu }\mathrm{g/ ml})=(12.25\times {A}_{663}-2.79\times {A}_{647}) \end{eqnarray*}

\begin{eqnarray*}\mathrm{Chlorophyll}~\mathrm{b}~(\mathrm{\mu }\mathrm{g/ ml})=(21.50\times {A}_{647}-5.10\times {A}_{663}) \end{eqnarray*}

\begin{eqnarray*}\mathrm{Carotenoid}~(\mathrm{\mu }\mathrm{g/ ml})=(1000\times {A}_{470}-1.82\mathrm{chlorophyll}~\mathrm{a}-85.02\mathrm{chlorophyll}~\mathrm{b})/198. \end{eqnarray*}

### Photosynthetic O_2_ evolution measurement

The Qubit Systems’ S104 Differential O_2_ Analyzer (DOX) was used to measure the photosynthetic O_2_ evolution. The chamber was calibrated using nitrogen gas and then oxygen (O_2_) was added to 21%. Leaf samples were placed into the chamber and ambient air was injected. The released oxygen from leaves was measured by the device sensor for 1000 s. The light was set at 66 µmol m^−2^s^−1^.

### Maximum quantum yield of PSII and relative chlorophyll level determination

Before removing plants from pots, chlorophyll concentration in the4thleavesof all plants was measured by a chlorophyll content meter (Hansatech Instruments, CL-01, UK). Maximum quantum yield of PSII (*F*_v_∕*F*_m_) (*F*_v_: variable fluorescence; *F*_m_: maximum fluorescence) measurement was determined before plants removal from pots by a plant efficiency analysis meter (Hansatech Instruments, Handy PEA, UK).

### Free amino acids, proline and glycine betaine content measurements

In order to evaluate the concentration of free amino acids, fresh sample was homogenized in 50 mM phosphate buffer (pH 6.8). The extracts were centrifuged for 20 min at 3,000 rpm then ninhydrin reagent was added to supernatant and heated for 7 min in water bath at 100 °C. Absorbance at 570 nm was determined immediately after cooling in the cold water bath. Different concentrations of glycine were used to make standard curve (Razavi, Nasrollahi & Ghasemian, 2017). To estimate proline content, 0.5 g of fresh leaf tissue was grounded in 10 ml sulfosalicylic acid. After addition of 2 ml ninhydrin reagent, 2 ml acetic acid and 2 ml toluene, two different phases appeared. Absorption of colored supernatant containing toluene and proline was measured at 520 nm. Standard curve was made using different concentrations of proline (Mahajan et al., 2018). For measuring glycine betaine concentration, 0.5 g of dried leaf tissues were grounded, homogenized with 20 ml deionized water and shaken for 24 h at 25 °C. After filtering the samples, they were diluted 1:1with 2N sulfuric acid, cooled for 1 h and 10 ml Lugol’s iodine was added. After vortexing, all samples were centrifuged and stored at 4 °C for 16 h. Residual crystals were dissolved in 1, 2-dichloroethane and the absorbance was measured at 365 nm. Standard curve was prepared by different concentrations of glycine betaine in 1N H_2_SO_4_ (Al Hassan et al., 2016).

### Hydrogen peroxide (H_2_**O**_2_) level measurement

For measurement of H_2_O_2_ content, fresh leaf tissues were ground on ice in a mortar with 1% trichloro acetic acid. Samples were centrifuged at 4 °C for 15 min at 10,000 g. 0.5 ml of supernatant was mixed with 0.5 ml of 10 mM phosphate buffer (pH 7) and 1 ml of 1 M potassium iodide. Content of H_2_O_2_ was measured spectrophotometrically at 390 nm. Different concentrations of H_2_O_2_ were used to make standard curve (Velikova, Yordanov & Edreva, 2000).

### Protein extraction

0.05 g of fresh leaf tissue was homogenized with 2 ml of 0.1 M phosphate buffer (pH 6.8). All extracts were centrifuged at 13,000 g for 15 min at 4 °C and used to measure the enzyme activities [19] and protein content. Protein concentration was estimated with Bradford reagent using bovine serum albumin as standard (Bradford, 1976).

### Catalase (CAT) activity measurement

In order to examine CAT activity, a reaction mixture including 2.5 ml of 0.05 M phosphate buffer (pH 7), 0.3 ml of 3% H_2_O_2_ and 0.2 ml protein extract was prepared. Enzyme activity was examined using Beer–Lambert’s method as H_2_O_2_ removal measured through decrease in absorbance at 240 nm (Aebi, 1984).

### Polyphenol oxidase (PPO) activity assessment

For PPO activity, the reaction mixture including 2.5 ml 0.2 M phosphate buffer (pH 6.8) and 0.2 ml of 0.02 M pyrogallol was incubated at 40 °C water bath. After adding 0.2 ml of the enzyme extract, absorbance changes were measured at 430 nm (Patra & Mishra, 1979). Enzyme activity was estimated by Beer-Lambert’s method.

### Ascorbate peroxidase (APX) activity measurement

In order to evaluate APX activity, the reaction mixture included 2.5 ml 0.2 M phosphate buffer (pH6.5), 0.2 ml of 3% H_2_O_2_, 5 mM ascorbic acid and 0.2 ml enzyme extract (Nakano & Asada, 1981). The APX activity was estimated by Beer-Lambert’s method at 290 nm.

### Protease activity determination

To estimate protease activity, 0.5 ml of 1% casein (provided in 0.5 M phosphate buffer, pH 6) with 0.2 ml enzyme extract were incubated at 45 °C for 1 h. The reaction was stopped by adding 0.1 ml of 40% trichloroacetic acid (TCA). Protease activity was calculated based on an extinction coefficient of 21.5 mM^−1^cm^−1^ andabsorbance measurement at 280 nm (Drapeau, 1974).

### Malondialdehyde (MDA) measurement

For MDA determination, 0.2 g fresh leaf tissues were ground in 5 ml of 0.1% TCA. After centrifuging at 4,000 rpm for 20 min, 2.5 ml 0.5% thiobarbituric acid (TBA) in 20% TCA were added to the supernatants. Extracts were incubated in water bath at 95 °C for 30 min and immediately cooled on ice. Afterwards, all samples were centrifuged at 4,000 rpm for 30 min and absorbance was measured at 532 nm and 600 nm. The MDA concentration was calculated by subtracting the non-specific absorption at 600 nm from the absorption at 532 nm using absorbance extinction coefficient of 155 mM^−1^cm^1^ (Sadak, Abdelhamid & El-Saady, 2010).

### Total betalain content

Betalain extraction was carried out by a modified method of Francis (2000). 5 g fresh leaves were ground in 25 ml of 20% ethanol and 0.5% citric acid solution and left in darkness for 24 h. After filtering, the supernatant was used for betalain purification by normal phase column chromatography. 10 g of silica gel 60packed with the binary solvent mixture of methanol/water 8:2 v/v with 1% v/v glacial acetic acid were used as stationary phase. The same binary solvent was applied as an elution at flow rate of 0.7 ml/min. The elution was used for spectrophotometric measurement of absorbance at 535 nm.

Total betalain content was estimated using following equation:

\begin{eqnarray*}\mathrm{Betalain}~\mathrm{content}~(\mathrm{mg}/100~\mathrm{gFW})= \frac{A.F.M}{\varepsilon } \cdot \frac{V}{1000 \mathrm{m}} \end{eqnarray*}

A: sample absorption

M: average molecular mass

ε: molar extinction coefficient

V: extract volume

m: mass of vegetable solid used for extraction

DF: dilution factor.

### Statistical analysis

Statistical analysis was carried out by one-way ANOVA (Version 18; SPSS Inc., Chicago, USA). Means compression were performed by Duncan Test at *P* < 0.05. Graphs were drawn in Excel Software (Microsoft Office, 2007).

## Results

### Growth Parameters and Relative Water Content

**Figure 1:**
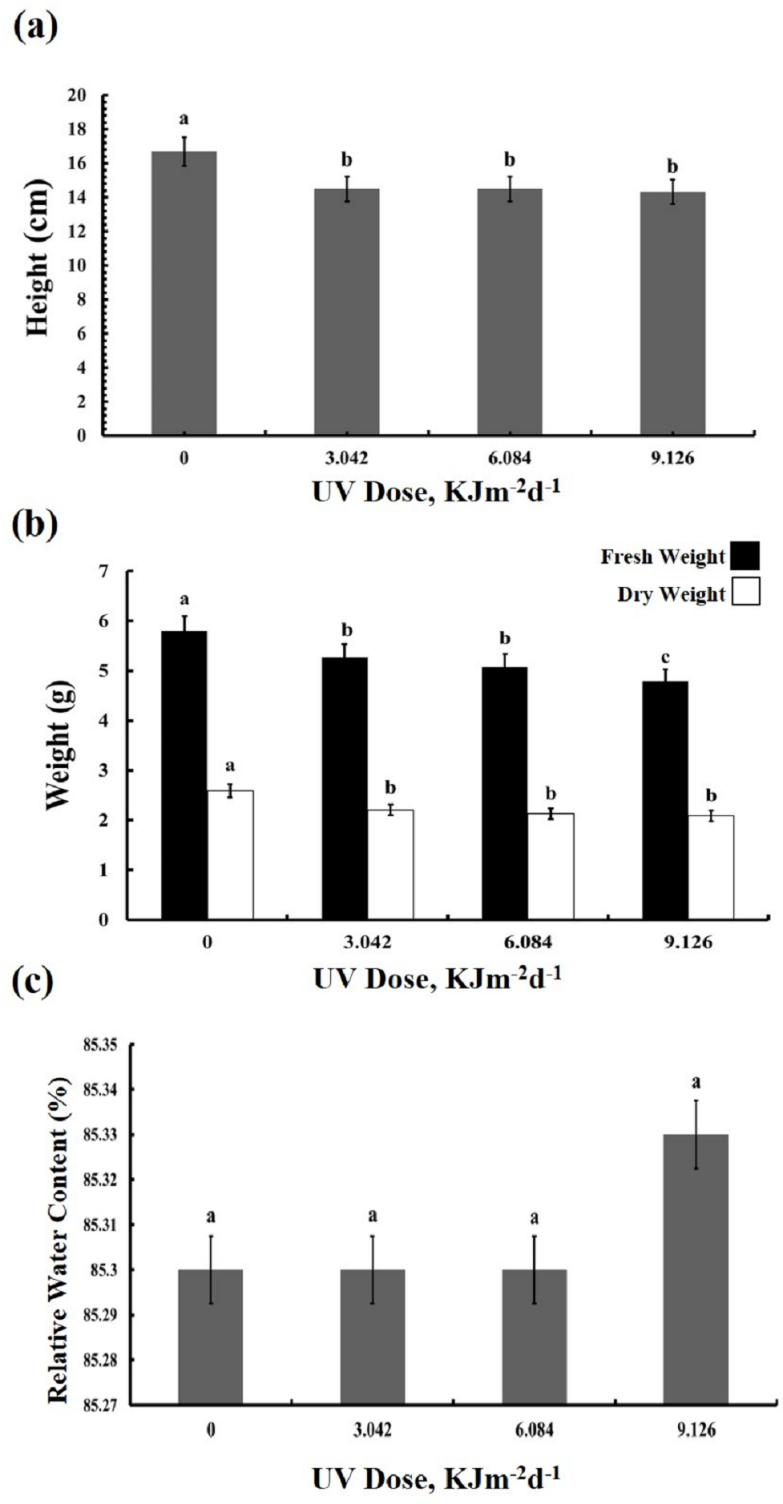
Effects of three different doses of UV-B radiation (3.042, 6.084 and 9.126 kJm^−2^*d*^−1^) on (A) plant height, (B) plant fresh and dry weight, and (C) relative water content.

The UV-B treated plants showed leaf chlorosis and a significant decrease in the plant’s height irrespective of the UV-B dose (Fig. 1A). Furthermore, UV-B irradiance caused reduction by 10–20% in both fresh and dry weight of leaves (Fig. 1B). The fresh weight of plants treated with the highest dose of UV-B showed the greatest decrease in fresh weight, whereas the weights of plants treated with the lowest and middle doses were identical. On the other hand, no dose-dependent difference in the dry weight of the three UV-B treated samples was observed. However, compared with the control, leaf relative water content was not affected in the UV treated plants (Fig. 1C). Thus, while UV-B treatment affected the growth of the sugar beet, a three-fold difference in dose of UV-B irradiation had only a minor influence on the BR1 cultivar (Dataset S1, Dataset S2, Dataset S3).

### Photosynthetic performance

**Figure 2:**
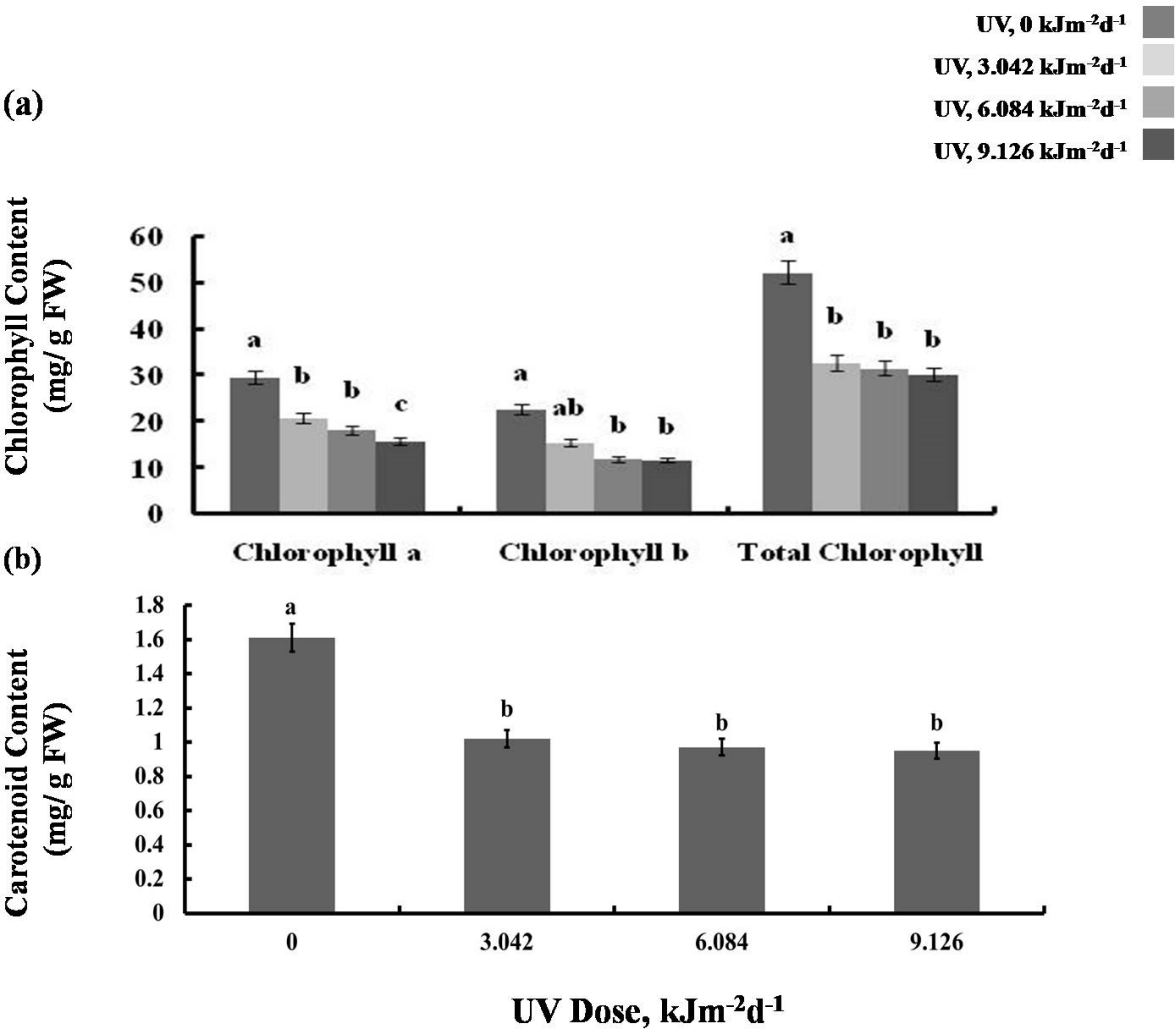
Effects of three different doses of UV-B radiation (3.042, 6.084 and 9.126 kJm^−2^*d*^−1^) on (A) chlorophyll, and (B) carotenoid content. Shown are means ± S.D. Different letters mark significantly different values for individual pigments at *P* < 0.05 (ANOVA).

**Figure 3:**
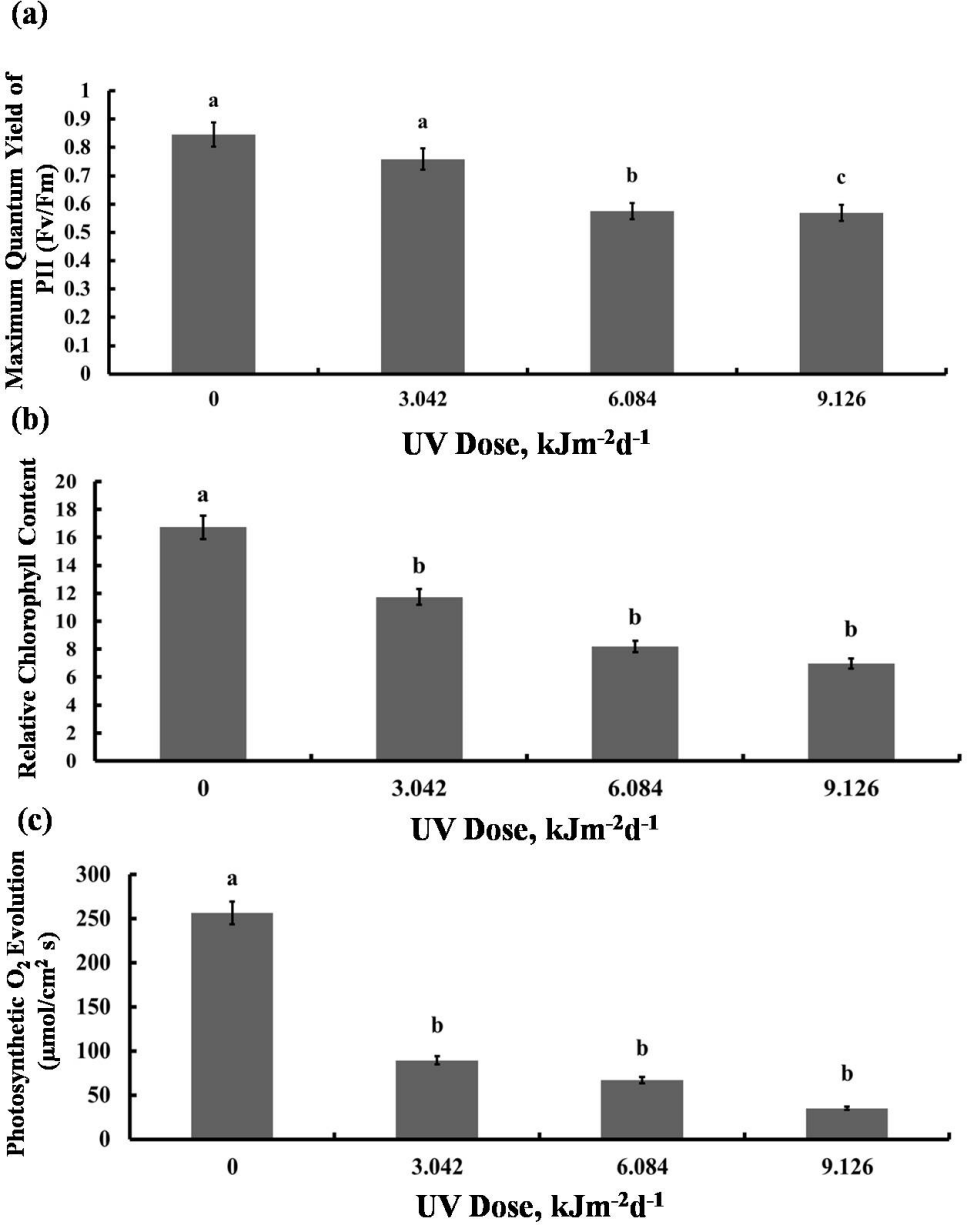
Effects of three different doses of UV-B radiation (3.042, 6.084 and 9.126 kJm^−2^*d*^−1^) on (A) Maximum quantum yield of PSII (Fv/Fm), (B) Relative chlorop hyll content and (C) photosynthesis O_2_ evolution. Shown are means ± S.D. Different letters mark significantly different values at *P* < 0.05 (ANOVA).

We then tested whether the reduction in growth is caused by decreased photosynthetic performance. The contents of photosynthetic pigments, chlorophyll a and b, as well as total chlorophyll, declined in all experimental groups to a similar degree (Fig. 2A). The same was true for carotenoid content which was reduced by 40% in all UV-B treated plants, compared to the control (Fig. 2B). However, compared with the control, photochemical efficiency of photosystem II (*F*_V_/ *F*_M_ index) declined only in the plants exposed to 6.084 and 9.126 kJm^−2^d^−1^ UV-B, by 28%, despite no difference in the content of pigments(Fig. 3A). On the other hand, the reduction in relative chlorophyll content (SPAD) in UV-B exposed plants was significant compared to control plants but not between the different UV-B doses (Fig. 3B).

Significant reduction in photosynthetic O_2_ evolution was observed in all plants exposed to UV-B compared with the control, with the plants treated with 9.126 kJm^−2^d^−1^UV-B releasing only 15% O_2_ compared to controls (Fig. 3C). Clearly, photosynthesis performance of sugar beet is greatly affected by UV-B, but the irradiation dose seems to have only a minor effect (Dataset S1, Dataset S2, Dataset S3).

### Metabolite Contents

**Figure 4:**
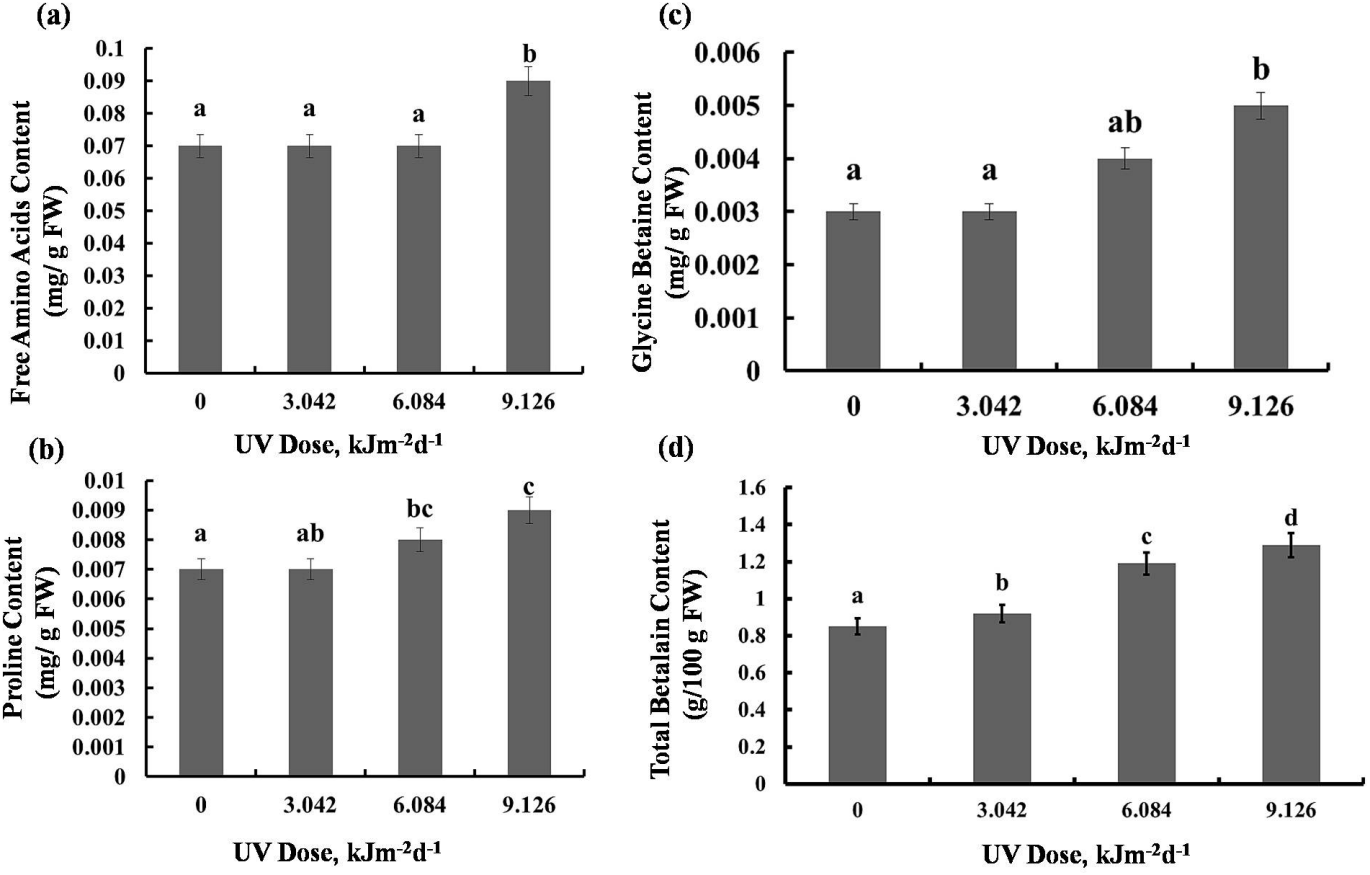
Effects of three different doses of UV-B radiation (3.042, 6.084 and 9.126 kJm^−2^*d*^−1^) on contents of (A) free amino acids, (B) proline, (C) glycine betaine and (D) total betalain. Shown are means ± S.D. Different letters mark significantly different values *P* < 0.05 (ANOVA).

There was maximum 22% increase in total free amino acids level in plants exposed to 9.126 kJm^−2^d^−1^UV-B. However, plants exposed to 3.042 and 6.084 kJm^−2^d ^−1^UV-B didn’t exhibit significant changes in free amino acids accumulation (Fig. 4A). Compared with the control, 3.042 kJm^−2^d^−1^ UV-B caused no significant change in proline content. However, 6.084 and 9.126 kJm^−2^d ^−1^ UV-B doses increased proline content by 24% and 29% respectively (Fig. 4B). The glycine betaine content was increased by 9.126 kJm^−2^d^−1^ UV-B, while 3.042 and 6.084 kJm^−2^d^−1^ UV-B did not cause significant changes in glycine betaine content in comparison with the control (Fig. 4C). As indicated in Fig. 4D, total betalain concentration increased in a dose dependent manner by 8%, 28% and 34% respectively, compared to the control (Dataset S1, Dataset S2, Dataset S3).

### Protein content and enzyme activities

**Figure 5:**
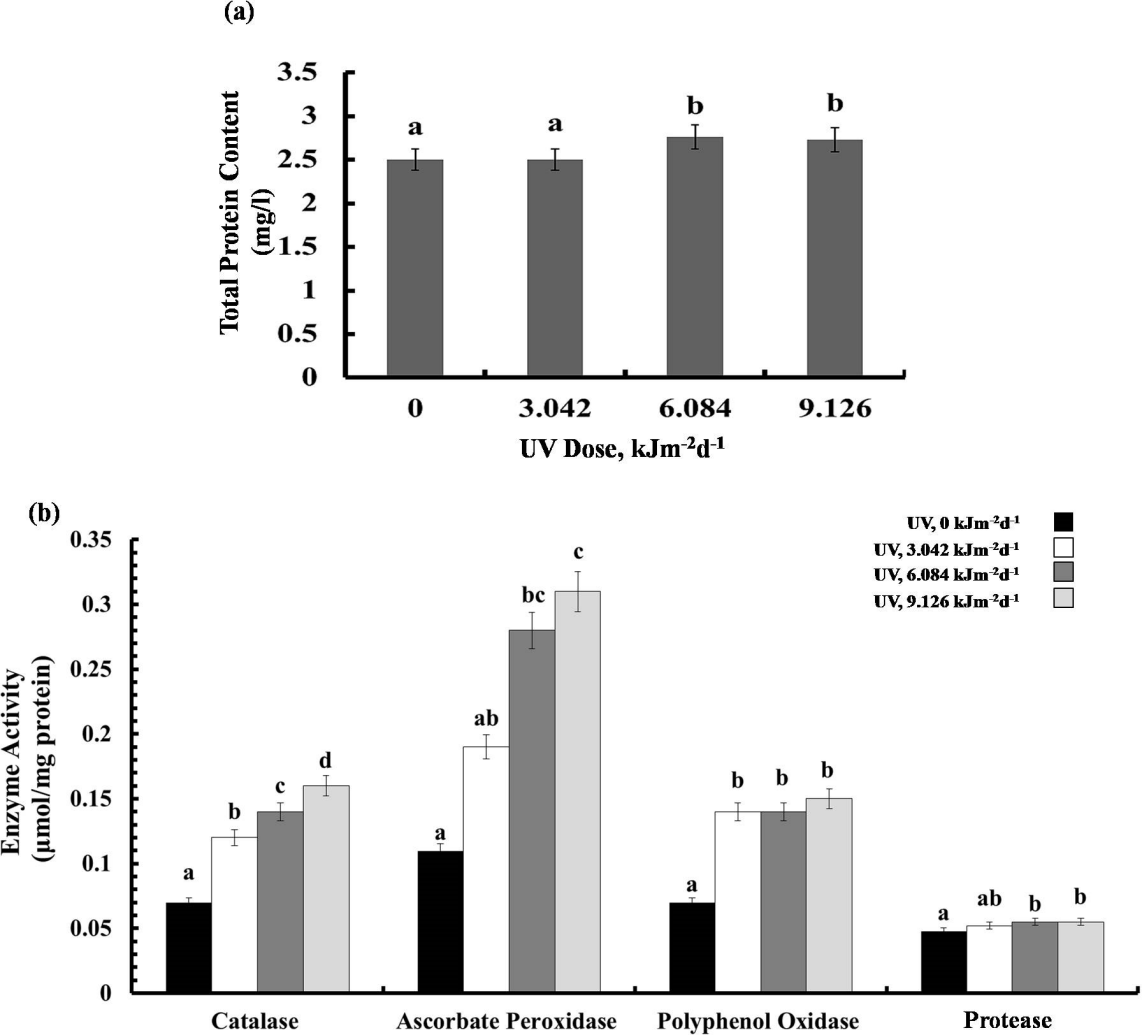
Effects of three different doses of UV-B radiation (3.042, 6.084 and 9.126 kJm^−2^*d*^−1^) on (A) leaf protein content, and (B) enzyme activities of catalase, ascorbate peroxidase, polyphenol oxidase and protease. Shown are means ± S.D. Different letters mark significantly different values *P* < 0.05 (ANOVA).

Exposure to 3.042 kJm^−2^d ^−1^UV-B did not affect protein content, however, plants treated with 6.084 and 9.126 kJm^−2^d ^−1^UV-B, showed 12% increase in protein concentration (Fig. 5A). Different UV-B doses caused dose dependent significant increase in CTA activity by 41%, 50%, and 56%, respectively (Fig. 5B). Similarly, a gradual increase in APX activity was observed (Fig. 5B). The activity of PPO was raised significantly in all UV-B exposed plants to the same extent, irrespective of the UV-B dose. Protease activity was increased significantly only in 9.126 kJm^−2^d^−1^ UV-B treatment, while plants under 3.042 and 6.084 kJm^−2^d ^−1^ UV-B did not show any differences in protease activity compared with the control (Fig. 5B). Thus, the increase in antioxidant enzyme activity in response to UV stress seems to contribute to the relatively similar damage of the plants at different UV-B doses (Dataset S1, Dataset S2, Dataset S3).

### MDA and H_2_*O*_2_ content

**Figure 6:**
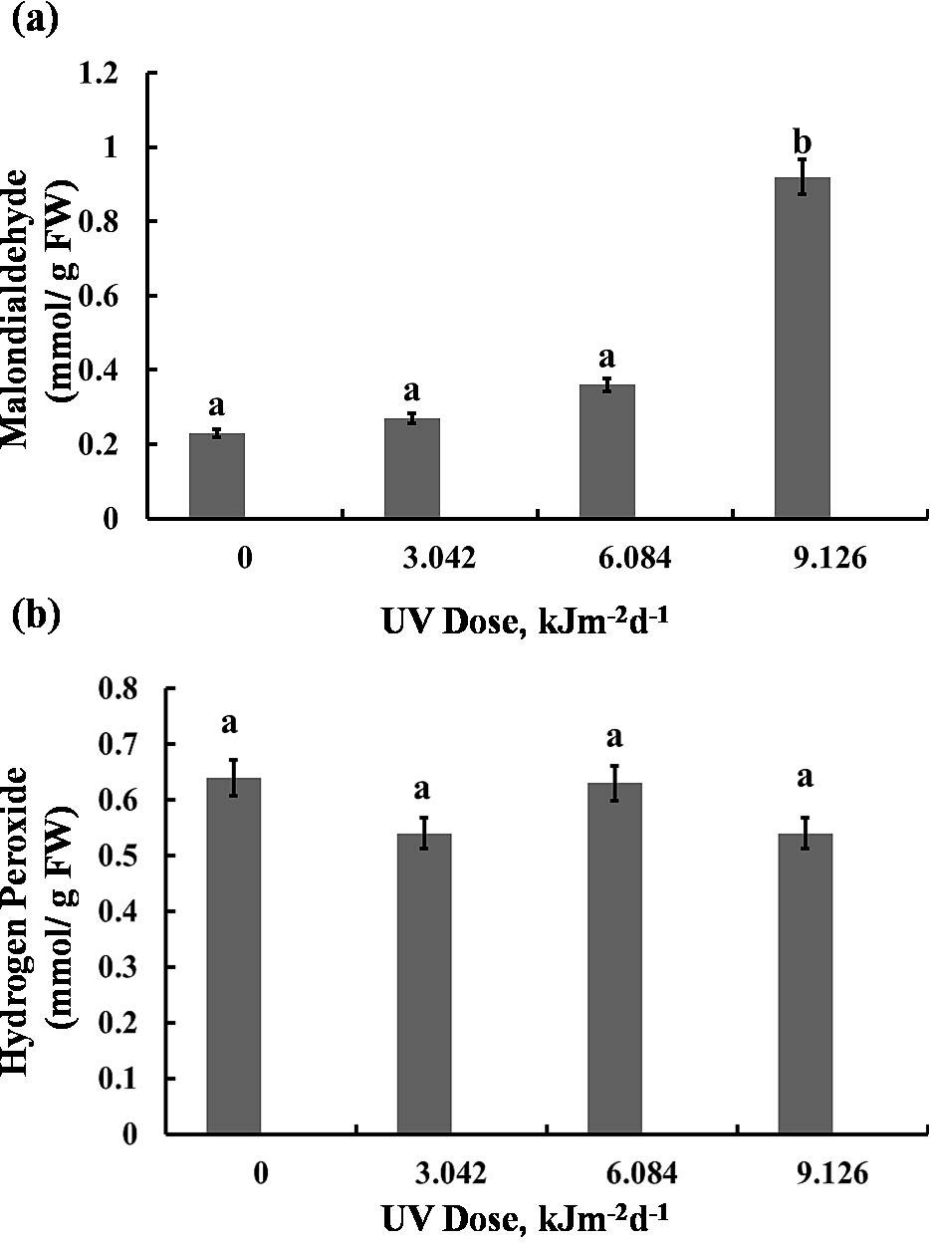
Effects of three different doses of UV-B radiation (3.042, 6.084 and 9.126 kJm^−2^*d*^−1^) on contents of (A) 1-malondialdehyde, and (B) hydrogen peroxide. Shown are means ±S.D. Different letters mark significantly different values *P* < 0.05 (ANOVA).

The highest UV-B dose caused a 75% increase in MDA content, however, lower doses (3.042 and 6.084 kJm^−2^d^−1^ UV-B) did not cause significant alterations (Fig. 6A). Interestingly, the H_2_O_2_ analyses demonstrated that UV-B did not cause any significant change in H_2_O_2_ levels compared with the control (Fig. 6B). Thus, the UV-B clearly led to oxidative stress, but not to accumulation of H_2_O_2_ (Dataset S1, Dataset S2, Dataset S3).

## Discussion

In the present work we studied the effects of high but realistic doses of UV-B on physiological parameters of young leaves of BR1 variety of sugar beet. According to Jalilian et al. (2009) BR1 in comparison to some other Iranian origin cultivars (7233, Shirin, Rasoul, 428 and 276), is the most freezing-resistant cultivar. Assadi Nassab et al. (2015) identified BR1 as a semi-tolerant cultivar to salt stress. They reported that salinity reduced shoot and root dry matter, leaf area, rate of photosynthesis (carbon dioxide assimilation) and leaf transpiration rate. As our results showed UV-B treatments resulted in a significant reduction in BR1 growth parameters, such as plant height, or dry and fresh weights. This reduction reached 10–20% (Fig. 1) and was thus significantly milder than the reduction in leaf area by 77% described previously (Khan, Kumar & Giridhar, 2016). In line with our findings, growth reduction under UV-B has also been reported in *Arabidopsis thaliana* (Morales et al., 2013) and *Capsicum annuum* (Rodriguez-Calzada et al., 2018). Besides, many other Iranian origin sugar beet cultivars (7221, 30973, IR7, Jolgeh, IC1 and 7233-P29) have shown growth reduction in response to various environmental stresses (Moosavi et al., 2017; Dadkhah, 2011).

Obviously, different UV-B doses have been used in the previous experiments that led to a too high level of stress. It was proposed previously that the growth inhibition in the UV-B treated sugar beet plants was caused by UV-promoted auxin degradation and photo-oxidation (Zhao, 2010). However, we revealed that UV-B caused a considerable reduction in photosynthesis rate, chlorophyll and carotenoid content and chlorophyll fluorescence (Figs. 2, 3), which is more likely to be responsible for the reduction in growth. The *F*_*v*_∕*F*_m_ value, as a chlorophyll fluorescence parameter, is a widely used measure for environmental stress evaluation. It represents the relative efficiency of electron transmission from PSII to PSI, as well as thylakoids membrane and photosystems integrity. Accordingly, PSII can be affected by environmental stresses leading to *F*_v_/*F*_m_ decrease. Fluorescence reduction in the present study is in agreement with those reported by Panagopoulos, Bornman & Bjorn (1990) and Clarke et al. (1995), who found that UV-B decreased sugar beet chlorophyll fluorescence. Reactive oxygen species (ROS) are the most common cause for reduction in photochemical efficiency and also photosynthesis rate. PSII reaction center is the most sensitive component of photosynthetic apparatus to UV-B exposure (Kataria, Jajoo & Guruprasad, 2014). The most common target of ROS in photosynthesis apparatus is the D1 protein, a part of the PSII reaction center complex. Indeed, Greenberg et al. (1989), reported that D1 degradation occurred rapidly in *Spirodela oligorrhiza* under UV radiation. The UV-B also reduces gene expression of chlorophyll a/b binding proteins that attach to chlorophylls in the structure of LHC in PSII. This leads to a disturbance in light harvesting process and photosynthesis inhibition (Doprikova & Apostolva, 2015). Since more oxygen is available in photosynthetic tissues to generate ROS, these tissues are more sensitive to UV radiation than non-photosynthetic ones. Moreover, UV radiation degrades Rubisco or inhibits its activity by affecting Rubisco activase. These effects are other possible causes for photosynthesis inhibition by UV-B. Furthermore, UV based stomata conductance regulation can also play an important role in photosynthesis reduction.

Our findings indicated that the UV-treated plants showed no change in leaf relative water content (Fig. 1C). This can be attributed to increasing some osmotic compatible solutes such as proline, glycine betaine, and other free amino acid in the leaves of irradiated sugar beet plants (Fig. 4). Relative water content is an important indicator of plant water status and it estimates the equilibrium between water potential and transpiration (Vesala et al., 2017). During environmental stresses, plants modify their metabolism in various ways including production of osmoregulatory compounds, such as proline and glycine betaine. Compatible solutes accumulate in the cytoplasm to control water potential equilibrium within the cell. Thus, their enhancement following UV-B treatment, can lead to normal relative water content which is vital for cell turgor and growth of plants. Indeed, in the current work, amount of proline and glycine betaine was increased at 6 and 9 kJm^−2^d ^−1^ of UV-B. This finding led us to the conclusion that the osmoregulation system of sugar beet plants is promoted only at relatively high doses of UV-B. Carbon used for the compatible solutes synthesis may represent up to 10% of total carbon, which may contribute to the growth and total biomass reduction in plants under UV-B radiation (Taiz & Zeiger, 2013). UV-B radiation led to remarkable increase in enzymatic antioxidant activities in sugar beet plants (Fig. 5B). Elevation of antioxidant enzymes activities is a defensive mechanism for UV radiation-induced ROS scavenging (Yadav & Sharma, 2016). CAT and peroxidase are the most important enzymes involved in H_2_O_2_ removal to protect plants against harmful effects of ROS on plant cell membranes and macromolecules. Accordingly, all doses of UV-B radiation increased CAT activity in this study (Fig. 5B). Willekens et al. (1995) proposed a classification of plant catalases: class I catalases play roles in photorespiration, class II catalases protect plants against environmental stresses and finally class III catalases are related to fatty acid degradation in glyoxysomes (Vesala et al., 2017). Based on this concept, we suggest that UV-B radiation induces class II catalases activity in *Beta vulgaris*. Moreover, APX activity was increased under 6.084 and 9.126 kJm^−2^d^−1^ of UV-B and all doses of UV-B caused a significant increase in PPO activity (Fig. 5B), confirming previous findings (Mahdavian, Ghorbanli & Kalantari, 2008; Kargarkhorrami, Jamei & Tabatabaei-Koupaei, 2014). Protease activity was increased only in plants treated with 9.126 kJm^−2^d^−1^ of UV-B. Presumably, a rise in protease activity of the UV irradiated plants can be a mechanism to break down storage proteins to produce free amino acids for osmotic adjustment and detoxification purposes. On the other hand, various isoforms of APX serve as H_2_O_2_ scavengers in cytosol and chloroplast. Majer et al. (2014) also found UV-B increased peroxidase activity in tobacco plants, although these plants did not exhibit catalase activity enhancement in response to UV radiation. In tobacco plants, peroxidase activity was accompanied by elevation of hydroxyl radical scavenging, so it seems that peroxidase is a strong antioxidant defense line in UV-B treated plants (Hollosy, 2002).

We have observed that UV-B radiation of sugar beet affected not only the enzyme activities but also the general protein accumulation. Inactivating different proteins and enzymes can be a consequence of aromatic amino acids photolysis by UV-B (Peykarestan et al., 2012). Also, UV-B can cause protein damage by impairing RNA. However, in this study 6 and 9 kJm^−2^d^−1^UV-B caused a significant increase in protein content. This increment may be ascribed to the increase in antioxidant enzymes or synthesis of heat-shock proteins. Heat-shock proteins or Stress-induced proteins are produced in cells that are exposed to different stresses. In fact, UV-B, like other stresses, can induce transcription of stress proteins (Al-Whaibi, 2011). However, in contrast to our finding, it has been shown that prolonged radiation of *Portulaca grandiflora* seeds with various doses of UV-B led to reduce in leaf protein content. In that case, however, the protein changes were observed in different tissues than those which were UV irradiated (Greenberg et al., 1989). Our findings also indicated that despite the high antioxidant enzyme activities, high dose of UV-B caused a significant increase in some stress markers such as MDA content in sugar beet plants. Raising the MDA contents is a characteristic sign for lipid peroxidation. It has been reported that thylakoid membranes with abundant unsaturated fatty acids can be peroxidized by stress-related ROS (Pospisil, 2016). Accordingly, thylakoid membranes peroxidation leads to membrane adhesion or disruption causing a photosynthesis rate reduction (Zancan et al., 2008). Interestingly, in our experiments with sugar beet UV-B did not cause any significant changes in H_2_O_2_ concentration. The high activities of CAT and APX thus seem to sufficiently prevent its accumulation, leading to similar H_2_O _2_ content in UV-B exposed sugar beet plants. Indeed, other studies concluded that H_2_O_2_ concentration in plants increases upon exposure to UV-B in addition to other stress factors such as salt stress (Zhao et al., 2015) and iron starvation (Zancan et al., 2008) more than after UV-B treatment alone. This phenomenon maybe explained by UV-B ability to photosensitize hydroxyl radical from H_2_O_2_ (Hideg, Jansen & Strid, 2013). Furthermore, it has been known that UV-B exposure leads to accumulation of UV-screening pigments and phytochemicals, including anthocyanins (Bjorn, Widell & Wang, 2002). Since in sugar beet betalains substitute the anthocyanins, we asked whether they also may have a role in UV-B protection in this species. Indeed, our findings showed that sugar beet plants increase their betalain concentration in response to all three UV-B doses (Fig. 4D). The increase in betalain concentration in response to stress has also been reported previously in *Riviniahumilis* berries. Khan, Kumar & Giridhar (2016) found in *Rivinia* berries treated with elicitors such as salicylic acid and chitosan, increased betalain accumulation, accompanied with CAT and SOD activation decrease, suggesting a possible ROS scavenging role for betalains. Increase in betalains due to UV-B (Fig. 4D), suggested for these water-soluble pigments a protecting function also against UV-B. Indeed, it has been previously indicated that betalains act as UV-protectors and ROS scavengers in ice plants (Ibdah et al., 2002).

## Conclusions

In conclusion, we assessed the effects of UV-B on sugar beet. Although growth and photosynthetic performance of BR1, evaluated cultivar, were negatively affected by UV, these effects were rarely dose dependent, which indicates a good tolerance of high UV irradiation doses. This was achieved by accumulation of betalain and proline, as well as induction of antioxidant enzymes. Thus, the BR1 cultivar seems to be suitable particularly for areas with high doses of UV-B irradiation.

##  Supplemental Information

10.7717/peerj.6790/supp-1 Dataset S1 Raw data

10.7717/peerj.6790/supp-2 Dataset S2 Processed data

10.7717/peerj.6790/supp-3 Dataset S3 Statistical analyses

## Additional Information and Declarations

### Data Availability

The following information was supplied regarding data availability: The raw data is available in a Supplemental File.
