# Supplementary material for: Physiological and biochemical responses of sugar beet (Beta vulgaris L) to ultraviolet-B radiation
Source: PeerJ. 2019 May 3;7:e6790. doi: 10.7717/peerj.6790 (PMC6501760; doi:10.7717/peerj.6790)
Supplement: Dataset S3 [file peerj-07-6790-s003.pdf]

```
ONEWAY Height FW DW RWC ChlorophyllA ChlorophyllB TotalChlorophyll Carotenoid
MaximumQuantumYeild RelativeChlorophyll PhotosynthesisRate Proline FreeAminoAs
ids GlycineBetaine protein CAT APX PPO Protease Malondeahdehyde H2O2 Betalain
BY Treatment
  /STATISTICS DESCRIPTIVES
  /MISSING ANALYSIS
  /POSTHOC=DUNCAN ALPHA(0.05).
```

Oneway

Notes

Output Created		25-Nov-2018 14:57:46
Comments		
Input	Data	C:\Users\Admin\Desktop\Untitled1.sav
	Active Dataset	DataSet1
	Filter	<none>
	Weight	<none>
	Split File	<none>
	N of Rows in Working Data File	12
Missing Value Handling	Definition of Missing	User-defined missing values are treated as missing.
	Cases Used	Statistics for each analysis are based on cases with no missing data for any variable in the analysis.
Syntax		ONEWAY Height FW DW RWC ChlorophyllA ChlorophyllB TotalChlorophyll Carotenoid MaximumQuantumYeild RelativeChlorophyll PhotosynthesisRate Proline FreeAminoAsids GlycineBetaine protein CAT APX PPO Protease Malondeahdehyde H2O2 Betalain BY Treatment /STATISTICS DESCRIPTIVES /MISSING ANALYSIS /POSTHOC=DUNCAN ALPHA (0.05).
Resources	Processor Time	00:00:01.935
	Elapsed Time	00:00:02.225

```
[DataSet1] C:\Users\Admin\Desktop\Untitled1.sav
```


created with nitro PDF professional
download the free trial online at nitropdf.com/professional

Descriptives

		N	Mean	Std. Deviation	Std. Error
Height	0	3	16.6333	.30551	.17638
	3.042	3	14.5000	.43589	.25166
	6.084	3	14.5667	.11547	.06667
	9.126	3	14.3667	.23094	.13333
	Total	12	15.0167	1.00980	.29150
FW	0	3	5.8033	.11676	.06741
	3.042	3	5.2000	.24269	.14012
	6.084	3	5.3800	.23388	.13503
	9.126	3	4.7933	.18009	.10398
	Total	12	5.2942	.41542	.11992
DW	0	3	2.5933	.08021	.04631
	3.042	3	2.2167	.03055	.01764
	6.084	3	2.1367	.08505	.04910
	9.126	3	2.0967	.08505	.04910
	Total	12	2.2608	.21496	.06205
RWC	0	3	.8533	.02517	.01453
	3.042	3	.8567	.03512	.02028
	6.084	3	.8500	.04359	.02517
	9.126	3	.8500	.04583	.02646
	Total	12	.8525	.03279	.00946
ChlorophyllA	0	3	29.5667	1.15180	.66499
	3.042	3	20.7500	.59573	.34395
	6.084	3	19.8800	.46033	.26577
	9.126	3	18.1333	1.20658	.69662
	Total	12	22.0825	4.68464	1.35234
ChlorophyllB	0	3	22.6933	9.13177	5.27223
	3.042	3	15.3533	2.35789	1.36133
	6.084	3	11.8467	2.02112	1.16690
	9.126	3	11.6533	.55734	.32178
	Total	12	15.3867	6.22504	1.79701
TotalChlorophyll	0	3	52.2667	8.25288	4.76480
	3.042	3	32.7133	2.98775	1.72498
	6.084	3	31.5700	1.52325	.87945
	9.126	3	30.1533	.27006	.15592
	Total	12	36.6758	10.18477	2.94009

created with nitro PDF professional
download the free trial online at nitropdf.com/professional

Descriptives

		95% Confidence Interval for Mean		Minimum	Maximum
		Lower Bound	Upper Bound		
Height	0	15.8744	17.3922	16.30	16.90
	3.042	13.4172	15.5828	14.20	15.00
	6.084	14.2798	14.8535	14.50	14.70
	9.126	13.7930	14.9404	14.10	14.50
	Total	14.3751	15.6583	14.10	16.90
FW	0	5.5133	6.0934	5.70	5.93
	3.042	4.5971	5.8029	5.00	5.47
	6.084	4.7990	5.9610	5.24	5.65
	9.126	4.3460	5.2407	4.67	5.00
	Total	5.0302	5.5581	4.67	5.93
DW	0	2.3941	2.7926	2.51	2.67
	3.042	2.1408	2.2926	2.19	2.25
	6.084	1.9254	2.3479	2.04	2.20
	9.126	1.8854	2.3079	2.00	2.16
	Total	2.1243	2.3974	2.00	2.67
RWC	0	.7908	.9158	.83	.88
	3.042	.7694	.9439	.82	.89
	6.084	.7417	.9583	.80	.88
	9.126	.7362	.9638	.80	.89
	Total	.8317	.8733	.80	.89
ChlorophyllA	0	26.7054	32.4279	28.79	30.89
	3.042	19.2701	22.2299	20.28	21.42
	6.084	18.7365	21.0235	19.41	20.33
	9.126	15.1360	21.1306	16.80	19.15
	Total	19.1060	25.0590	16.80	30.89
ChlorophyllB	0	.0088	45.3779	15.11	32.83
	3.042	9.4960	21.2107	13.68	18.05
	6.084	6.8259	16.8674	10.64	14.18
	9.126	10.2688	13.0379	11.01	11.99
	Total	11.4315	19.3419	10.64	32.83
TotalChlorophyll	0	31.7654	72.7680	46.01	61.62
	3.042	25.2914	40.1353	29.27	34.62
	6.084	27.7860	35.3540	30.43	33.30
	9.126	29.4825	30.8242	29.88	30.42
	Total	30.2047	43.1469	29.27	61.62

created with nitroPDF professional
download the free trial online at nitropdf.com/professional

Descriptives

		N	Mean	Std. Deviation	Std. Error
Carotenoid	0	3	1.6100	.22539	.13013
	3.042	3	1.0267	.07572	.04372
	6.084	3	.9533	.05033	.02906
	9.126	3	.9767	.06658	.03844
	Total	12	1.1417	.30343	.08759
MaximumQuantumYeild	0	3	.8460	.02524	.01457
	3.042	3	.7593	.07022	.04054
	6.084	3	.6437	.09349	.05398
	9.126	3	.5533	.01528	.00882
	Total	12	.7006	.12708	.03668
RelativeChlorophyll	0	3	16.7300	.30512	.17616
	3.042	3	11.7600	.15395	.08888
	6.084	3	8.8333	.15275	.08819
	9.126	3	6.9333	.15275	.08819
	Total	12	11.0642	3.86365	1.11534
PhotosynthesisRate	0	3	256.2600	7.31931	4.22581
	3.042	3	89.5500	1.57724	.91062
	6.084	3	67.1100	1.07093	.61830
	9.126	3	34.9500	1.64082	.94733
	Total	12	111.9675	89.40209	25.80816
Proline	0	3	.00700	.002646	.001528
	3.042	3	.00700	.002646	.001528
	6.084	3	.00900	.003606	.002082
	9.126	3	.01300	.001732	.001000
	Total	12	.00900	.003464	.001000
FreeAminoAsids	0	3	.07540	.003974	.002294
	3.042	3	.07850	.002689	.001552
	6.084	3	.07483	.003482	.002010
	9.126	3	.09367	.003512	.002028
	Total	12	.08060	.008535	.002464
GlycineBetaine	0	3	.00300	.001732	.001000
	3.042	3	.00333	.003215	.001856
	6.084	3	.00633	.002082	.001202
	9.126	3	.00800	.001000	.000577
	Total	12	.00517	.002855	.000824

created with nitro PDF professional
download the free trial online at nitropdf.com/professional

Descriptives

		95% Confidence Interval for Mean		Minimum	Maximum
		Lower Bound	Upper Bound		
Carotenoid	0	1.0501	2.1699	1.35	1.75
	3.042	.8386	1.2148	.94	1.08
	6.084	.8283	1.0784	.90	1.00
	9.126	.8113	1.1421	.92	1.05
	Total	.9489	1.3345	.90	1.75
MaximumQuantumYeild	0	.7833	.9087	.83	.87
	3.042	.5849	.9338	.68	.81
	6.084	.4114	.8759	.58	.75
	9.126	.5154	.5913	.54	.57
	Total	.6198	.7813	.54	.87
RelativeChlorophyll	0	15.9720	17.4880	16.52	17.08
	3.042	11.3776	12.1424	11.59	11.89
	6.084	8.4539	9.2128	8.70	9.00
	9.126	6.5539	7.3128	6.80	7.10
	Total	8.6093	13.5190	6.80	17.08
PhotosynthesisRate	0	238.0778	274.4422	248.25	262.60
	3.042	85.6319	93.4681	87.74	90.63
	6.084	64.4497	69.7703	65.98	68.11
	9.126	30.8740	39.0260	33.61	36.78
	Total	55.1641	168.7709	33.61	262.60
Proline	0	.00043	.01357	.005	.010
	3.042	.00043	.01357	.004	.009
	6.084	.00004	.01796	.006	.013
	9.126	.00870	.01730	.011	.014
	Total	.00680	.01120	.004	.014
FreeAminoAsids	0	.06553	.08527	.071	.079
	3.042	.07182	.08518	.077	.082
	6.084	.06618	.08348	.071	.078
	9.126	.08494	.10239	.090	.097
	Total	.07518	.08602	.071	.097
GlycineBetaine	0	-.00130	.00730	.002	.005
	3.042	-.00465	.01132	.001	.007
	6.084	.00116	.01150	.004	.008
	9.126	.00552	.01048	.007	.009
	Total	.00335	.00698	.001	.009

created with nitro PDF professional
download the free trial online at nitropdf.com/professional

Descriptives

		N	Mean	Std. Deviation	Std. Error
protein	0	3	2.5000	.24556	.14177
	3.042	3	2.5000	.25632	.14799
	6.084	3	2.9933	.04163	.02404
	9.126	3	2.9433	.07371	.04256
	Total	12	2.7342	.29047	.08385
CAT	0	3	.0753	.00929	.00536
	3.042	3	.1217	.00961	.00555
	6.084	3	.1440	.00458	.00265
	9.126	3	.1693	.00379	.00219
	Total	12	.1276	.03663	.01057
APX	0	3	.1160	.00500	.00289
	3.042	3	.1997	.10704	.06180
	6.084	3	.2853	.02838	.01638
	9.126	3	.3147	.01474	.00851
	Total	12	.2289	.09412	.02717
PPO	0	3	.0797	.00814	.00470
	3.042	3	.1420	.02784	.01607
	6.084	3	.1477	.00231	.00133
	9.126	3	.1597	.00757	.00437
	Total	12	.1323	.03485	.01006
Protease	0	3	.0487	.00404	.00233
	3.042	3	.0527	.00416	.00240
	6.084	3	.0550	.00265	.00153
	9.126	3	.0553	.00153	.00088
	Total	12	.0529	.00394	.00114
Malondeahdehyde	0	3	.2367	.00577	.00333
	3.042	3	.2700	.11790	.06807
	6.084	3	.3667	.08327	.04807
	9.126	3	.9233	.07024	.04055
	Total	12	.4492	.29822	.08609
H2O2	0	3	.6400	.28618	.16523
	3.042	3	.5467	.16258	.09387
	6.084	3	.6333	.33828	.19531
	9.126	3	.5433	.42525	.24552
	Total	12	.5908	.27510	.07942

created with nitro PDF professional
download the free trial online at nitropdf.com/professional

Descriptives

		95% Confidence Interval for Mean			
		Lower Bound	Upper Bound	Minimum	Maximum
protein	0	1.8900	3.1100	2.23	2.71
	3.042	1.8633	3.1367	2.26	2.77
	6.084	2.8899	3.0968	2.96	3.04
	9.126	2.7602	3.1264	2.86	3.00
	Total	2.5496	2.9187	2.23	3.04
CAT	0	.0523	.0984	.07	.09
	3.042	.0978	.1455	.11	.13
	6.084	.1326	.1554	.14	.15
	9.126	.1599	.1787	.17	.17
	Total	.1043	.1509	.07	.17
APX	0	.1036	.1284	.11	.12
	3.042	-.0662	.4656	.13	.32
	6.084	.2148	.3558	.26	.32
	9.126	.2780	.3513	.30	.33
	Total	.1691	.2887	.11	.33
PPO	0	.0594	.0999	.07	.09
	3.042	.0728	.2112	.12	.17
	6.084	.1419	.1534	.14	.15
	9.126	.1409	.1785	.15	.17
	Total	.1101	.1544	.07	.17
Protease	0	.0386	.0587	.05	.05
	3.042	.0423	.0630	.05	.06
	6.084	.0484	.0616	.05	.06
	9.126	.0515	.0591	.05	.06
	Total	.0504	.0554	.05	.06
Malondeahdehyde	0	.2223	.2510	.23	.24
	3.042	-.0229	.5629	.17	.40
	6.084	.1598	.5735	.30	.46
	9.126	.7489	1.0978	.85	.99
	Total	.2597	.6386	.17	.99
H2O2	0	-.0709	1.3509	.34	.91
	3.042	.1428	.9505	.42	.73
	6.084	-.2070	1.4737	.25	.89
	9.126	-.5130	1.5997	.11	.96
	Total	.4160	.7656	.11	.96

created with nitro PDF professional
download the free trial online at nitropdf.com/professional

Descriptives

		N	Mean	Std. Deviation	Std. Error
Betalain	0	3	.8500	.07937	.04583
	3.042	3	.9667	.03055	.01764
	6.084	3	1.1900	.05568	.03215
	9.126	3	1.4600	.06557	.03786
	Total	12	1.1167	.24861	.07177

Descriptives

		95% Confidence Interval for Mean			
		Lower Bound	Upper Bound	Minimum	Maximum
Betalain	0	.6528	1.0472	.79	.94
	3.042	.8908	1.0426	.94	1.00
	6.084	1.0517	1.3283	1.13	1.24
	9.126	1.2971	1.6229	1.40	1.53
	Total	.9587	1.2746	.79	1.53

ANOVA

		Sum of Squares	df	Mean Square
Height	Between Groups	10.517	3	3.506
	Within Groups	.700	8	.087
	Total	11.217	11	
FW	Between Groups	1.579	3	.526
	Within Groups	.319	8	.040
	Total	1.898	11	
DW	Between Groups	.465	3	.155
	Within Groups	.044	8	.005
	Total	.508	11	
RWC	Between Groups	.000	3	.000
	Within Groups	.012	8	.001
	Total	.012	11	
ChlorophyllA	Between Groups	234.706	3	78.235
	Within Groups	6.699	8	.837
	Total	241.404	11	
ChlorophyllB	Between Groups	239.574	3	79.858
	Within Groups	186.689	8	23.336

created with nitro PDF professional
download the free trial online at nitropdf.com/professional

ANOVA

		F	Sig.
Height	Between Groups	40.063	.000
	Within Groups		
	Total		
FW	Between Groups	13.185	.002
	Within Groups		
	Total		
DW	Between Groups	28.374	.000
	Within Groups		
	Total		
RWC	Between Groups	.021	.996
	Within Groups		
	Total		
ChlorophyllA	Between Groups	93.436	.000
	Within Groups		
	Total		
ChlorophyllB	Between Groups	3.422	.073
	Within Groups		

created with nitroPDF professional
download the free trial online at nitropdf.com/professional

ANOVA

		Sum of Squares	df	Mean Square
ChlorophyllB	Total	426.262	11	
TotalChlorophyll	Between Groups	982.164	3	327.388
	Within Groups	158.860	8	19.857
	Total	1141.024	11	
Carotenoid	Between Groups	.886	3	.295
	Within Groups	.127	8	.016
	Total	1.013	11	
MaximumQuantumYeild	Between Groups	.149	3	.050
	Within Groups	.029	8	.004
	Total	.178	11	
RelativeChlorophyll	Between Groups	163.879	3	54.626
	Within Groups	.327	8	.041
	Total	164.206	11	
PhotosynthesisRate	Between Groups	87800.281	3	29266.760
	Within Groups	119.798	8	14.975
	Total	87920.080	11	
Proline	Between Groups	.000	3	.000
	Within Groups	.000	8	.000
	Total	.000	11	
FreeAminoAsids	Between Groups	.001	3	.000
	Within Groups	.000	8	.000
	Total	.001	11	
GlycineBetaine	Between Groups	.000	3	.000
	Within Groups	.000	8	.000
	Total	.000	11	
protein	Between Groups	.662	3	.221
	Within Groups	.266	8	.033
	Total	.928	11	
CAT	Between Groups	.014	3	.005
	Within Groups	.000	8	.000
	Total	.015	11	
APX	Between Groups	.072	3	.024
	Within Groups	.025	8	.003

created with nitroPDF professional
download the free trial online at nitropdf.com/professional

ANOVA

		F	Sig.
ChlorophyllB	Total		
TotalChlorophyll	Between Groups	16.487	.001
	Within Groups		
	Total		
Carotenoid	Between Groups	18.599	.001
	Within Groups		
	Total		
MaximumQuantumYeild	Between Groups	13.622	.002
	Within Groups		
	Total		
RelativeChlorophyll	Between Groups	1336.695	.000
	Within Groups		
	Total		
PhotosynthesisRate	Between Groups	1954.401	.000
	Within Groups		
	Total		
Proline	Between Groups	3.200	.084
	Within Groups		
	Total		
FreeAminoAsids	Between Groups	19.836	.000
	Within Groups		
	Total		
GlycineBetaine	Between Groups	3.738	.060
	Within Groups		
	Total		
protein	Between Groups	6.626	.015
	Within Groups		
	Total		
CAT	Between Groups	89.302	.000
	Within Groups		
	Total		
APX	Between Groups	7.722	.010
	Within Groups		

created with nitro PDF professional
download the free trial online at nitropdf.com/professional

ANOVA

		Sum of Squares	df	Mean Square
APX	Total	.097	11	
PPO	Between Groups	.012	3	.004
	Within Groups	.002	8	.000
	Total	.013	11	
Protease	Between Groups	.000	3	.000
	Within Groups	.000	8	.000
	Total	.000	11	
Malondeahdehyde	Between Groups	.927	3	.309
	Within Groups	.052	8	.006
	Total	.978	11	
H2O2	Between Groups	.025	3	.008
	Within Groups	.807	8	.101
	Total	.832	11	
Betalain	Between Groups	.651	3	.217
	Within Groups	.029	8	.004
	Total	.680	11	

ANOVA

		F	Sig.
APX	Total		
PPO	Between Groups	17.033	.001
	Within Groups		
	Total		
Protease	Between Groups	2.633	.122
	Within Groups		
	Total		
Malondeahdehyde	Between Groups	47.891	.000
	Within Groups		
	Total		
H2O2	Between Groups	.084	.967
	Within Groups		
	Total		
Betalain	Between Groups	59.280	.000
	Within Groups		
	Total		

Post Hoc Tests

created with nitro PDF professional
download the free trial online at nitropdf.com/professional

Homogeneous Subsets

Height

Duncan[a]

Treatment	N	Subset for alpha = 0.05	
		1	2
9.126	3	14.3667	
3.042	3	14.5000	
6.084	3	14.5667	
0	3		16.6333
Sig.		.450	1.000

Means for groups in homogeneous subsets are displayed.

a. Uses Harmonic Mean Sample Size = 3.000.

FW

Duncan[a]

Treatment	N	Subset for alpha = 0.05		
		1	2	3
9.126	3	4.7933		
3.042	3		5.2000	
6.084	3		5.3800	
0	3			5.8033
Sig.		1.000	.302	1.000

Means for groups in homogeneous subsets are displayed.

a. Uses Harmonic Mean Sample Size = 3.000.

DW

Duncan[a]

Treatment	N	Subset for alpha = 0.05	
		1	2
9.126	3	2.0967	
6.084	3	2.1367	
3.042	3	2.2167	
0	3		2.5933
Sig.		.093	1.000

Means for groups in homogeneous subsets are displayed.

a. Uses Harmonic Mean Sample Size = 3.000.

created with nitroPDF professional
download the free trial online at nitropdf.com/professional

RWC

Duncan[a]

Treatment	N	Subset for alpha = 0.05
		1
6.084	3	.8500
9.126	3	.8500
0	3	.8533
3.042	3	.8567
Sig.		.845

Means for groups in homogeneous subsets are displayed.

a. Uses Harmonic Mean Sample Size = 3.000.

ChlorophyllA

Duncan[a]

Treatment	N	Subset for alpha = 0.05		
		1	2	3
9.126	3	18.1333		
6.084	3		19.8800	
3.042	3		20.7500	
0	3			29.5667
Sig.		1.000	.278	1.000

Means for groups in homogeneous subsets are displayed.

a. Uses Harmonic Mean Sample Size = 3.000.

ChlorophyllB

Duncan[a]

Treatment	N	Subset for alpha = 0.05	
		1	2
9.126	3	11.6533	
6.084	3	11.8467	
3.042	3	15.3533	15.3533
0	3		22.6933
Sig.		.394	.100

Means for groups in homogeneous subsets are displayed.

a. Uses Harmonic Mean Sample Size = 3.000.

created with nitro PDF professional
download the free trial online at nitropdf.com/professional

TotalChlorophyll

Duncan[a]

Treatment	N	Subset for alpha = 0.05	
		1	2
9.126	3	30.1533	
6.084	3	31.5700	
3.042	3	32.7133	
0	3		52.2667
Sig.		.519	1.000

Means for groups in homogeneous subsets are displayed.

a. Uses Harmonic Mean Sample Size = 3.000.

Carotenoid

Duncan[a]

Treatment	N	Subset for alpha = 0.05	
		1	2
6.084	3	.9533	
9.126	3	.9767	
3.042	3	1.0267	
0	3		1.6100
Sig.		.513	1.000

Means for groups in homogeneous subsets are displayed.

a. Uses Harmonic Mean Sample Size = 3.000.

MaximumQuantumYeild

Duncan[a]

Treatment	N	Subset for alpha = 0.05	
		1	2
9.126	3	.5533	
6.084	3	.6437	
3.042	3		.7593
0	3		.8460
Sig.		.104	.116

Means for groups in homogeneous subsets are displayed.

a. Uses Harmonic Mean Sample Size = 3.000.

created with nitro PDF professional
download the free trial online at nitropdf.com/professional

RelativeChlorophyll

Duncan[a]

Treatment	N	Subset for alpha = 0.05			
		1	2	3	4
9.126	3	6.9333			
6.084	3		8.8333		
3.042	3			11.7600	
0	3				16.7300
Sig.		1.000	1.000	1.000	1.000

Means for groups in homogeneous subsets are displayed.

a. Uses Harmonic Mean Sample Size = 3.000.

PhotosynthesisRate

Duncan[a]

Treatment	N	Subset for alpha = 0.05			
		1	2	3	4
9.126	3	34.9500			
6.084	3		67.1100		
3.042	3			89.5500	
0	3				256.2600
Sig.		1.000	1.000	1.000	1.000

Means for groups in homogeneous subsets are displayed.

a. Uses Harmonic Mean Sample Size = 3.000.

Proline

Duncan[a]

Treatment	N	Subset for alpha = 0.05	
		1	2
0	3	.00700	
3.042	3	.00700	
6.084	3	.00900	.00900
9.126	3		.01300
Sig.		.416	.111

Means for groups in homogeneous subsets are displayed.

a. Uses Harmonic Mean Sample Size = 3.000.

created with nitro PDF professional
download the free trial online at nitropdf.com/professional

FreeAminoAsids

Duncan[a]

Treatment	N	Subset for alpha = 0.05	
		1	2
6.084	3	.07483	
0	3	.07540	
3.042	3	.07850	
9.126	3		.09367
Sig.		.246	1.000

Means for groups in homogeneous subsets are displayed.

a. Uses Harmonic Mean Sample Size = 3.000.

GlycineBetaine

Duncan[a]

Treatment	N	Subset for alpha = 0.05	
		1	2
0	3	.00300	
3.042	3	.00333	
6.084	3	.00633	.00633
9.126	3		.00800
Sig.		.107	.372

Means for groups in homogeneous subsets are displayed.

a. Uses Harmonic Mean Sample Size = 3.000.

protein

Duncan[a]

Treatment	N	Subset for alpha = 0.05	
		1	2
0	3	2.5000	
3.042	3	2.5000	
9.126	3		2.9433
6.084	3		2.9933
Sig.		1.000	.746

Means for groups in homogeneous subsets are displayed.

a. Uses Harmonic Mean Sample Size = 3.000.

created with nitro PDF professional
download the free trial online at nitropdf.com/professional

CAT

Duncan[a]

Treatment	N	Subset for alpha = 0.05			
		1	2	3	4
0	3	.0753			
3.042	3		.1217		
6.084	3			.1440	
9.126	3				.1693
Sig.		1.000	1.000	1.000	1.000

Means for groups in homogeneous subsets are displayed.

a. Uses Harmonic Mean Sample Size = 3.000.

APX

Duncan[a]

Treatment	N	Subset for alpha = 0.05		
		1	2	3
0	3	.1160		
3.042	3	.1997	.1997	
6.084	3		.2853	.2853
9.126	3			.3147
Sig.		.104	.097	.539

Means for groups in homogeneous subsets are displayed.

a. Uses Harmonic Mean Sample Size = 3.000.

PPO

Duncan[a]

Treatment	N	Subset for alpha = 0.05	
		1	2
0	3	.0797	
3.042	3		.1420
6.084	3		.1477
9.126	3		.1597
Sig.		1.000	.205

Means for groups in homogeneous subsets are displayed.

a. Uses Harmonic Mean Sample Size = 3.000.

created with nitro PDF professional
download the free trial online at nitropdf.com/professional

Protease

Duncan[a]

Treatment	N	Subset for alpha = 0.05	
		1	2
0	3	.0487	
3.042	3	.0527	.0527
6.084	3	.0550	.0550
9.126	3		.0553
Sig.		.053	.367

Means for groups in homogeneous subsets are displayed.

a. Uses Harmonic Mean Sample Size = 3.000.

Malondeahdehyde

Duncan[a]

Treatment	N	Subset for alpha = 0.05	
		1	2
0	3	.2367	
3.042	3	.2700	
6.084	3	.3667	
9.126	3		.9233
Sig.		.094	1.000

Means for groups in homogeneous subsets are displayed.

a. Uses Harmonic Mean Sample Size = 3.000.

H2O2

Duncan[a]

Treatment	N	Subset for alpha = 0.05
		1
9.126	3	.5433
3.042	3	.5467
6.084	3	.6333
0	3	.6400
Sig.		.734

Means for groups in homogeneous subsets are displayed.

a. Uses Harmonic Mean Sample Size = 3.000.

created with nitroPDF professional
download the free trial online at nitropdf.com/professional

Betalain

Duncan[a]

Treatment	N	Subset for alpha = 0.05			
		1	2	3	4
0	3	.8500			
3.042	3		.9667		
6.084	3			1.1900	
9.126	3				1.4600
Sig.		1.000	1.000	1.000	1.000

Means for groups in homogeneous subsets are displayed.

a. Uses Harmonic Mean Sample Size = 3.000.

created with nitro PDF professional
download the free trial online at nitropdf.com/professional
